# DRAPA trial – closed-suction drains versus closed gravity drains in pancreatic surgery: study protocol for a randomized controlled trial

**DOI:** 10.1186/s13063-015-0706-1

**Published:** 2015-05-07

**Authors:** Filip Čečka, Martin Loveček, Bohumil Jon, Pavel Skalický, Zdeněk Šubrt, Alexander Ferko

**Affiliations:** Department of Surgery, Faculty of Medicine, University Hospital Hradec Králové, Sokolská 581, 500 05 Hradec Králové, Czech Republic; First Department of Surgery, Faculty of Medicine, University Hospital Olomouc, IP Pavlova 6, 779 00 Olomouc, Czech Republic; Department of Field Surgery, Faculty of Military Health Sciences, University of Defence, Třebešská 1575, 500 02 Hradec Králové, Czech Republic

**Keywords:** Closed-suction drain, Distal pancreatectomy, Pancreatic fistula, Pancreaticoduodenectomy, Passive gravity drain

## Abstract

**Background:**

The morbidity of pancreatic resection remains high, with pancreatic fistula being the most common cause. The important question is whether any postoperative treatment adjustment may prevent the development of clinically significant postoperative pancreatic fistulae. Recent studies have shown that intraabdominal drains and manipulation using them are of great importance. Although authors of a few retrospective reports have described good results of pancreatic resection without the use of intraabdominal drains, a recent prospective randomized trial showed that routine elimination of drains in pancreaticoduodenectomy is associated with poor outcome. An important issue arises as to which type of drain is most suitable for pancreatic resection. Two types of surgical drains exist: open drains and closed drains. Open drains are considered obsolete nowadays because of frequent retrograde infection. Closed drains include two types: passive gravity drains and closed-suction drains. Closed-suction drains are more effective, as they remove fluid from the abdominal cavity under light pressure. However, some surgeons believe that closed-suction drains represent a potential hazard to patients and that negative pressure might increase the risk of pancreatic fistulae. Nobody has yet specifically dealt with the question of which kind of drainage is most appropriate in pancreatic surgery.

**Methods/Design:**

The aim of the DRAins in PAncreatic surgery (DRAPA) trial is to compare the closed-suction drain versus the closed passive gravity drain in pancreatic resection. DRAPA is a dual-centre, prospective, randomized controlled trial. The primary endpoint is the rate of postoperative pancreatic fistula; the secondary endpoint is postoperative morbidity with follow-up of 3 months.

**Discussion:**

No study to date has compared different types of drains in pancreatic surgery. This study is designed to answer the question whether any particular type of drain might lower the rate of postoperative pancreatic fistula or other complications.

**Trial registration:**

ClinicalTrials.gov Identifier: NCT01988519. Registered 13 November 2013.

## Background

Pancreatic resection is the only potentially curative method for treatment of pancreatic neoplasms. These surgical procedures are technically demanding, with pancreaticoduodenectomy and distal pancreatectomy being the most common [1]. Although the mortality rate associated with these procedures has dropped to under 5% in recent decades, the morbidity remains high [2,3]. The most significant and ominous complication is the postoperative pancreatic fistula (POPF) [4]. Although this complication is in most cases not life-threatening, it prolongs the hospital stay and requires additional treatment (for example with antibiotics, nutritional support) and interventions (such as endoscopy, interventional radiology and/or reoperation) and thus increases the cost of the treatment [2,5]. According to the International Study Group of Pancreatic Fistula (ISGPF) consensus definition, pancreatic fistula is diagnosed if the amylase content in fluid from the intraabdominal drains exceeds three times the normal serum concentration [6].

Several methods have been studied in the past with the goal of lowering the pancreatic fistula rate. They include pharmacological prophylaxis with octreotide [7] and various technical modifications of pancreatic remnant management after pancreaticoduodenectomy [8,9] and after distal pancreatectomy [10]. However, the use of octreotide remains controversial, and none of the studied techniques proved to be superior.

Another method of influencing the rate of pancreatic fistulae and postoperative complications is the use of intraabdominal drains and manipulation with them. Recent studies show that the use of drains, the type of drain and the manipulation with the drain can lower the pancreatic fistula rate [11-13].

Intraabdominal drains have traditionally been used in surgical fields [14]. The main significance of the drains is to prevent formation of intraabdominal fluid collection; furthermore, it helps with early diagnosis of postoperative bleeding, pancreatic and biliary leak, or anastomotic dehiscence [15-17]. In some cases, keeping the drain in place for a longer period of time can be part of conservative treatment of the pancreatic fistula, such as by creating a controlled pancreaticocutaneous fistula until the fistula has healed spontaneously without any additional treatment [11,18].

Recent studies show that the use of drains might not be beneficial for the patient, such as in cholecystectomy, appendicectomy, gastric resection, bowel resection and liver resection [19]. Moreover, the use of drains might even be harmful for the patient, as it can slow down recovery and the restoration of bowel movements and can prolong the hospital stay or even cause postoperative complications such as retrograde intraabdominal infection, hollow organ perforation and/or loss of fluid and electrolytes.

Pancreatic surgery is different from surgery of hollow organs. Unlike enteric anastomotic dehiscence, which often presents with pneumoperitoneum and frequently causes peritonitis [20], pancreatic leak is more frequent, but the clinical course is not usually so dramatic [4]. Pancreatic leak or pancreatic fistulae can be easily diagnosed by analysing the amylase concentration in the drain effluent [21]. However, the amylase concentration is increased in the majority of patients on the first postoperative day, even in those patients who do not develop pancreatic fistulae in the postoperative course. This means that the pancreatic anastomosis is not ‘watertight’ in the majority of the patients [22].

The important question is whether any treatment adjustment in the postoperative course may prevent the development of clinically significant POPF. Recent studies have shown that intraabdominal drains and the manipulation with them are of great importance [11,13,23]. Three important questions arise when studying drains in pancreatic surgery:

1. Is it necessary to use drains following pancreatic resection?
2. When should the drains be removed?
3. Which type of intraabdominal drain is most suitable?

These questions are addressed in turn in the subsections below.

### Is it necessary to use drains following pancreatic resection?

Drains have traditionally been used in abdominal surgery in most procedures, including pancreatic resection. The first pilot study describing the results of pancreaticoduodenectomy in 22 patients without drains was published in 1992 [24]. A number of retrospective cohort studies have shown superior results of pancreatic resection without routine intraabdominal drainage [13], but only two randomized studies compared pancreatic resection with versus without drains. The first randomized controlled trial was conducted in Memorial Sloan Kettering Cancer Center in New York City [14]. It failed to show that surgically placed intraabdominal drains reduce the rate of complications.

In a recent study, Van Buren *et al*. showed level I evidence that elimination of routine drainage in all cases of pancreaticoduodenectomy results in unacceptably poor outcomes, and thus this approach cannot be recommended [25]. Further studies are needed to confirm these results.

### When should the drains be removed?

Two studies have addressed this question. In one retrospective study, researchers compared removal of the drains on day 4 versus day 8 [26]; in the other, investigators in a prospective randomized trial compared removal of drains on day 3 versus after day 5 in patients with low risk of POPF [15]. Both studies showed a lower rate of intraabdominal complications and fluid collections, and a lower rate of POPF, in the group with early drain removal.

### Which type of intraabdominal drain is most suitable?

The final issue regarding drains in pancreatic surgery is which type of drain to use. Two types of surgical drains exist: open drains and closed drains. Open drains are considered obsolete because their use is associated with frequent retrograde infection [27]. Closed drains include two types: passive gravity drains and closed-suction drains. Generally speaking, closed-suction drains are preferred in the United States [28,29], and passive gravity drains are used more frequently in Europe [30]. Only two studies have compared the different types of drains: the Penrose drain versus closed-suction drain [31,32]. However, these two studies were retrospective and conducted over a long period of time (17 years and 22 years, respectively); comparison of the drains was not the primary endpoint; and the studies’ results are contradictory. Nobody has specifically dealt with the question of which kind of drainage is more appropriate in pancreatic surgery [12]. Diener *et al*., in a systematic review of the current state of evidence for drains in pancreatic surgery, stated that the role of different types of drains remains unclear [11].

## Methods/Design

### Objectives and hypothesis

The aim of the DRAins in PAncreatic surgery (DRAPA) trial is to compare two types of intraabdominal drains in pancreatic resection and their effect on the development of POPF and postoperative complications. Closed-suction drains are more effective, as they remove fluid from the abdominal cavity under light negative pressure [13]. However, some surgeons believe that closed-suction drains represent a potential hazard to patients [33] and that negative pressure might increase the risk of POPF [13].

The following hypotheses will be tested:

- H0: The risk of development of POPF and postoperative complications is equal in both groups.
- H1: The risk of development of POPF and postoperative complications is different between the groups.

### Study population and eligibility criteria

The DRAPA trial will encompass two participating centres: University Hospital Hradec Králové and University Hospital Olomouc, both in the Czech Republic. All patients who are scheduled for pancreatic resection at the two participating centres will be screened and assessed for eligibility.

Only patients who undergo pancreaticoduodenectomy or distal pancreatic resection will be included in the study. Patients with nonstandard procedures or a procedure associated with higher morbidity will be excluded from the study to achieve a homogeneous study group. Detailed inclusion and exclusion criteria are described below.

#### Inclusion criteria

1. Patients scheduled for primary pancreaticoduodenectomy or distal pancreatic resection in participating centres
2. Aged 18 years or older
3. Signed informed consent provided

#### Exclusion criteria

1. No pancreatic resection performed: nonresectable tumour
2. Total pancreatectomy, central pancreatectomy or enucleation
3. Multivisceral resection
4. Laparoscopic procedure
5. Resection of the portal vein and reconstruction with a graft
6. Lack of compliance, informed consent not provided or refusal to participate

### Sample size calculation

The sample size calculation is based on the expected rate of POPF from our previous experience, as there exist no pilot data comparing the closed-suction drain and closed passive gravity drain in pancreatic surgery. The POPF rate in the closed passive gravity drain group is expected to be 35% based on previous studies [2,3]. The POPF rate in the closed-suction drain group is expected to drop to half that rate (17.5%). The sample size calculation is based on a one-sided *t*-test for differences with respect to the primary endpoint, which is the POPF rate. With α= 5% and β = 20%, a sample size of 97 patients per group is necessary to detect a difference between the groups. With an expected dropout rate of 15%, we plan to enrol 223 patients into the study.

### Ethics, study registration and consent

This trial was approved by independent ethics committees at both participating institutions: the University Hospital Hradec Králové (registration number 201308 S27P) and the University Hospital Olomouc (registration number 129/13). The DRAPA trial will be conducted in the context of Good Clinical Practice and in accordance with the Declaration of Helsinki. The trial is registered at ClinicalTrials.gov under the registration number NCT01988519. All patients who are scheduled for pancreatic resection in participating institutions will be screened for eligibility and informed in detail about the DRAPA trial. Informed consent will be obtained from each participant. The study procedures, risks, benefits and data management will be clarified with the patients before they are asked to give their informed consent to participate.

### Study treatment

The surgical technique is standardized in both participating institutions and has been described previously [2]. Standard resection of the pancreatic head with duodenum is performed, followed by the reconstruction phase. A jejunum limb is prepared and brought through the transverse mesocolon for the pancreatic and biliary anastomosis. Pancreaticojejunostomy is performed in end-to-side fashion. No stents are used. No additional manipulation, such as fibrin glue or reinforcement with meshes, is allowed. The end-to-side hepaticojejunostomy is made in a single layer of interrupted stitches about 15 cm distal from the pancreaticojejunostomy. End-to-side duodenojejunostomy is performed about 50 cm distally in the antecolic position. For distal pancreatectomy, after exploration of the abdominal cavity, the pancreas is exposed and transected by scalpel in a fish mouth fashion. A splenectomy is performed according to the surgeon’s discretion based on the benign or malignant nature of the tumour. The main pancreatic duct is occluded with a crossing stitch. The pancreatic remnant is then closed with interrupted stitches. In pancreaticoduodenectomy, two drains are placed: one in front of and one behind the pancreatic anastomosis. In distal pancreatectomy, one drain is placed near the pancreatic remnant. A second drain is placed in the left subphrenic area only when splenectomy is performed.

In patients assigned to the closed passive gravity drainage group, passive tube drains (pfm medical, Cologne, Germany) are used. In patients assigned to the closed-suction drain group, BLAKE silicone drains (Ethicon Endo-Surgery, Cincinnati, OH, USA) are used. Drains are pulled through a separate stab incision outside the laparotomy and fixed with a stitch to the skin. The volume of the fluid is measured every 24 hours and noted in the patient’s record form. Amylase content in the drain fluid is analysed every other day starting on the third postoperative day until removal of the drain. Drains are removed between day 4 and day 6 after the surgery if no POPF develops.

### Safety aspects

Pancreatic resection is a highly technically demanding procedure. High-volume surgeons with great experience have better results than low-volume surgeons with less experience [34]. In order to avoid bias based on the learning curve of the surgeons, every surgical procedure will be performed or supervised by a senior surgeon who has experience with at least 50 pancreatic resections. Insertion of both types of drain is a simple common procedure performed on a routine basis, and no special training is necessary and no complications are expected.

### Postoperative data collection

A daily visit of the study patients will be made by clinical investigators or a delegated physician. All protocol-required information collected during the trial will be entered into the patient’s record form. Preoperative data gathered include patient sex, age, body mass index, American Society of Anesthesiologists physical status classification system score and comorbidities, especially those considered to increase the risk of POPF (diabetes mellitus and ischaemic heart disease) [35,36]. Intraoperative data to be collected include surgery duration, estimated blood loss, diameter of the main pancreatic duct and pancreatic gland texture. The diameter of the main pancreatic duct will be measured with a set of probes. Postoperative data to be gathered include volume of the drain output measured every 24 hours, amylase content analysed every other day starting on day 3, and the day of drain removal. Laboratory tests will include serum amylase, C-reactive protein, bilirubin and leukocyte count on days 3 and 7 [37]. Postoperative course assessments will include duration of intensive care, hospital stay including readmissions for postoperative complications, reinterventions (reoperations, endoscopy and interventional radiology procedures) and the reasons for readmissions. All the patients will have undergo ultrasonography or computed tomography (CT) before discharge from the hospital to detect any fluid collections. The patients will be seen by a clinical investigator 6 weeks and 3 months after the surgery in the outpatient department.

### Primary and secondary endpoints

The primary endpoint of this study is the rate of POPF occurrence. POPF is defined by the ISGPF as any measurable volume of drain fluid on or after postoperative day 3 with amylase content greater than three times the normal upper serum value [6]. Three grades of POPF are determined according to clinical severity: A, B and C.

Grade A POPF, also called ‘transient fistula’, has no clinical impact. The patient’s clinical condition is good, and little or no change in clinical management is required. Hospital discharge is not delayed. The drains are usually removed within 3 weeks.

Grade B POPF is symptomatic and clinically apparent, and peripancreatic collections may occur. Changes in clinical management are required, such as nutritional support, as well as antibiotics in cases of infection, and new drains into the collections may be inserted. Hospital stay is usually delayed, or readmission may be required.

Grade C POPF is severe and clinically significant. The clinical condition of the patient is poor, and stability may be borderline. Major changes in clinical management are required, such as intensive care, CT-guided drainage of peripancreatic collections or reoperation.

The secondary endpoint is the postoperative morbidity, including wound infection, intraabdominal collections, delayed gastric emptying, postoperative haemorrhage, pneumonia, abdominal rupture, cardiac events and neurological complications (Table 1), as previously defined [5,30,38-41]. Postoperative complications are graded based on severity according to the Clavien-Dindo definition as modified by DeOliveira and colleagues for pancreatic surgery [42,43] (Table 2).

**Table 1:** **Clinical parameters and postoperative complications for analysis** ^**a**^

**Parameters**	**Definitions**
Hospital stay	Days from initial operation to hospital discharge plus any readmission within 30 days
Operating time	Time from skin incision to wound closure (minutes)
Delayed gastric emptying	Failure to resume solid diet with prolonged need for nasogastric tube as defined by ISGPS [40]
Biliary leak	Bilirubin concentration in the drain fluid at least three times the serum bilirubin concentration as defined by ISGLS [38]
Postoperative haemorrhage	Evidence of blood loss from drains and/or nasogastric tube, based on ultrasonography, as defined by ISGPS [41]
Intraabdominal fluid collection	Collection of fluid measuring ≥3 cm associated with clinical or laboratory abnormalities
Symptomatic fluidothorax	Fluid in the pleural cavity associated with respiratory distress or a need to evacuate the fluid
Abdominal rupture	Dehiscence of abdominal closure with need for resuture of the laparotomy during the initial hospital stay
Myocardial infarction	Increase of serum concentration of CK-MB and troponin and/or the following ECG changes: new Q waves ≥0.04 in duration, new persistent ST elevation and/or depression
Pneumonia	Presence of a new infiltrate on chest X-ray, as well as the following: body temperature >38°C, abnormal elevation of WBC, or positive sputum, and requiring antibiotic treatment
Acute renal failure	Serum creatinine >3.0 mg/dl and/or need for dialysis
Wound infection	Surgical site infection associated with laparotomy that develops during the initial hospital stay
Urinary tract infection	Culture-positive urine, pyuria or bacteriuria on urinalysis requiring antibiotic treatment

^a^CK-MB, Creatine kinase MB isoenzyme; ECG, Electrocardiogram; ISGLS, International Study Group of Liver Surgery; ISGPS, International Study Group for Pancreatic Surgery; WBC, White blood cells.

**Table 2:** **Complication grades according to the Dindo-DeOliveira classification scheme** ^**a**^

**Grade**	**Definition**
Grade I	Any deviation from the normal postoperative course without the need for pharmacological treatment or surgical, endoscopic and radiologic intervention
Grade II	Requiring pharmacological treatment with drugs other than those allowed for grade I complications
Grade III	Requiring surgical, endoscopic or radiological intervention
Grade IIIa	Intervention not under general anaesthesia
Grade IIIb	Intervention under general anaesthesia
Grade IV	Life-threatening complications requiring intensive care unit management
Grade IVa	Single-organ dysfunction
Grade IVb	Multiorgan dysfunction
Grade V	Death of patient

^a^The Dindo-DeOliveira classification system is reported in detail elsewhere [42,43].

### Methods for avoiding bias

#### Minimizing systemic bias

**Figure 1:**
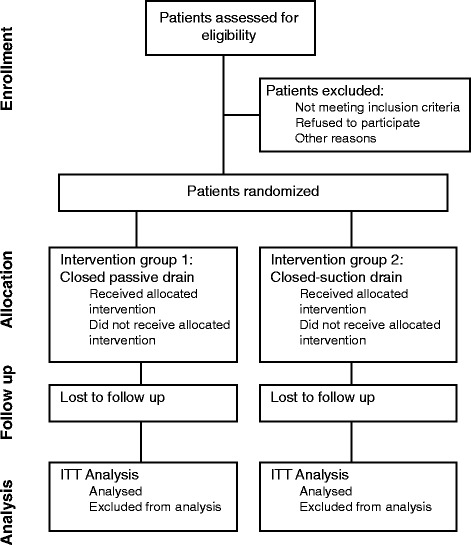
Flowchart of the process of the phases of the randomized trial according to the CONSORT guidelines.

Patients will be randomized to one of the drain groups during the surgical procedure after resectability of the tumour is confirmed. Randomization will be accomplished using balanced permutation blocks by generation of random numbers in order to obtain homogeneity between groups. Patients scheduled for pancreaticoduodenectomy and distal pancreatectomy will be randomized separately, as these two procedures are different with regard to the course and consequences of POPF [25,44]. Opaque, sealed envelopes will be produced, labelled with the randomization number and containing a sheet that states the group allocation for the patient. Randomization envelopes will be used in consecutive order. Basic characteristics of the patient and the day of randomization will be documented on a data sheet so that compliance to the randomization scheme may be checked retrospectively. If patients are excluded from the study after randomization, their numbers will not be reused. Obviously, operating surgeons, attending physicians and nursing staff cannot be blinded, as the drains look different. However, outcome assessors will be blinded. The randomization process will follow the CONSORT guidelines (Figure 1) [45].

#### Minimizing treatment bias

Pancreatic resection is standardized in both participating centres. Both types of drain are used in the participating departments; all surgeons participating in the study are familiar with them. Insertion of both types of drain is a simple, common procedure performed on a routine basis, which eliminates a learning curve.

#### Minimizing measurement bias

Detection and grading of POPF and postoperative complications, which are the primary and secondary endpoints, will be based on data in the patient’s record form. Blinding is not necessary, because the occurrence of POPF is an objective endpoint that cannot be influenced by the patient. Physician blinding is not possible, because the drains are apparently different.

### Statistical methods

Each patient’s allocation to the analysed population will be defined prior to the analysis and will be documented. In the full-analysis set, patients will be analysed as randomized according to the intention-to-treat principle. The intention-to-treat principle implies that the analysis includes all randomized patients. The per-protocol analysis set will include all the patients without major protocol deviation. Deviations from the protocol will be assessed as major or minor. Patients with major deviations from the protocol will be excluded from the per-protocol analysis. The safety analysis set will analyse patients according to the treatment.

The null hypothesis assumes that the risk of development of POPF and postoperative complications is equal in both groups. A binary logistic regression will be applied in order to compare POPF in the groups adjusting for other factors. Two-tailed *t*-tests or nonparametric Mann–Whitney *U* tests will be applied for continuous secondary endpoints. χ^2^ tests or Fisher’s exact test will be used for analysis of categorical secondary endpoints. A *P*-value <0.05 will be considered statistically significant. Statistical analyses will be performed using NCSS statistical software (NCSS, Kaysville, UT, USA).

## Discussion

Despite growing evidence in favour of pancreatic resection without intraabdominal drainage [13], routine elimination of drains cannot be recommended [25]. Rather more, drains could diminish the rate and severity of POPF in patients with high risk of POPF. Drains could possibly be avoided in patients with low risk of POPF [46]. However, the majority of pancreatic surgeons still consider routine placement of intraabdominal drains mandatory [15-17]. Once a decision is made to use intraabdominal drains, a controversy remains about which type of drain is most suitable in pancreatic resection [47]. Two types of drains are commonly used nowadays: passive gravity drains [26,30] and closed-suction drains [28,29]. Closed-suction drains are more effective, as they remove fluid from the abdominal cavity under light negative pressure [13]. However, some surgeons believe that closed-suction drains represent a potential hazard to patients [33] and that negative pressure might increase the risk of POPF [13]. No study conducted to date has specifically addressed the question of which type of drain is most beneficial for patients [12,13].

The DRAPA trial is designed to compare closed-suction drains with passive gravity drains in pancreatic surgery. Using the ISGPF definition of POPF will allow us to compare the results of this trial with those of other trials of drains in pancreatic surgery. This trial can show whether closed-suction drains represent hazards to patients or are beneficial in terms of decreasing POPF rates.

## Trial status

The DRAPA trial is currently recruiting patients. The last patient is expected to be recruited by December 2015.

## Abbreviations

CK-MB: Creatine kinase MB fraction
CONSORT: Consolidated Standards of Reporting Trials
DRAPA: DRAins in PAncreatic surgery trial
ECG: Electrocardiogram
ISGLS: International Study Group of Liver Surgery
ISGPF: International Study Group of Pancreatic Fistula
ISGPS: International Study Group of Pancreatic Surgery
POPF: Postoperative pancreatic fistula
WBC: White blood cells
